# Metabolic Control of Cardiomyocyte Cell Cycle

**DOI:** 10.14797/mdcvj.1309

**Published:** 2023-11-16

**Authors:** Ivan Menendez-Montes, Daniel J. Garry, Jianyi (Jay) Zhang, Hesham A. Sadek

**Affiliations:** 1 University of Texas Southwestern Medical Center, Dallas, Texas, US; 2 University of Minnesota, Minneapolis, Minnesota, US; 3 University of Alabama Birmingham, Birmingham, Alabama, US

**Keywords:** glycolysis, oxidative phosphorylation, cardiomyocyte proliferation, reactive oxygen species, uridine diphosphate N-acetylglucosamine (UDP GlycNAC), cardiac regeneration

## Abstract

Current therapies for heart failure aim to prevent the deleterious remodeling that occurs after MI injury, but currently no therapies are available to replace lost cardiomyocytes. Several organisms now being studied are capable of regenerating their myocardium by the proliferation of existing cardiomyocytes. In this review, we summarize the main metabolic pathways of the mammalian heart and how modulation of these metabolic pathways through genetic and pharmacological approaches influences cardiomyocyte proliferation and heart regeneration.

## Introduction

**Figure 1:**
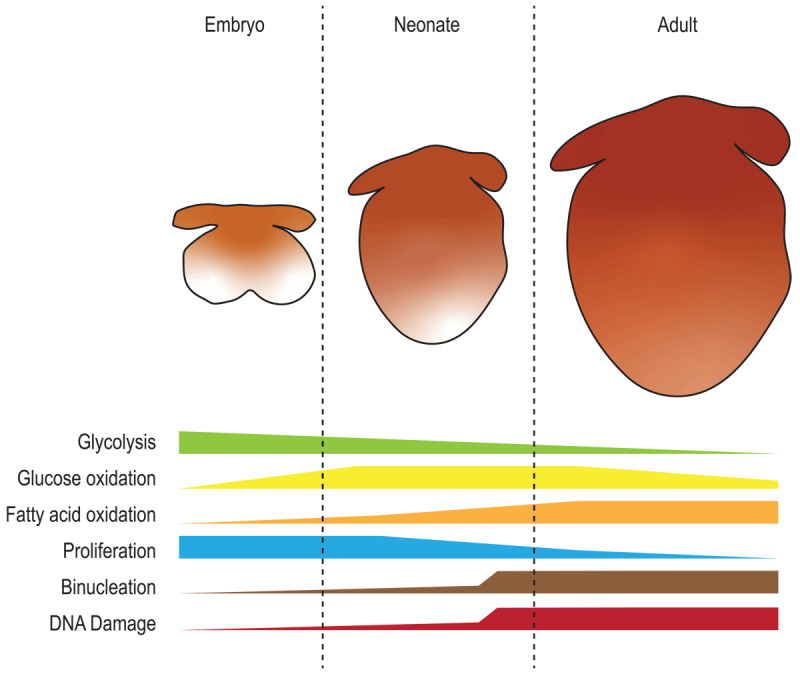
**Cardiac metabolism from embryo to adult.** Schematic representation of glycolysis, glucose oxidation, and fatty acid oxidation during embryonic, neonatal, and adult stages. The graph also includes the temporal dynamic of cardiac key processes (proliferation, binucleation, and DNA damage) in relation to the metabolic changes.

Heart failure is one of the leading causes of death in the western world. The pathophysiological basis of heart failure lies in the limited ability of the adult mammalian heart to regenerate damaged myocardium after an injury, such as a myocardial infarction (MI). Current therapies for heart failure aim to prevent the deleterious remodeling that occurs after MI injury, but there are currently no therapies to replace lost cardiomyocytes. However, several organisms, including adult zebrafish and neonatal mice, can regenerate their myocardium by the proliferation of existing cardiomyocytes.^1^ Interestingly, mammalian neonatal proliferation and regenerative potential is lost shortly after birth.^1,2^ This is linked to a neonatal metabolic switch in cardiomyocytes, where fatty acid oxidation increases at expenses of glucose oxidation and anaerobic glycolysis^3,4^ in order to support the huge adenosine triphosphatase (ATP) needs for contraction of the adult mammalian heart. This increase in mitochondrial oxidation of fatty acids results in increased reactive oxygen species (ROS) production, increased DNA damage and activation of the DNA damage response that ultimately leads to cell cycle exit and loss of cardiomyocyte proliferation (Figure 1).^5^

In addition to supporting ATP production, metabolism is involved in several processes in the heart and plays an important role in cardiomyocyte proliferation and heart regeneration. Moreover, several cardiac pathologies such as hypertrophy and diabetic cardiomyopathy, display alterations in the metabolic networks within the cardiomyocyte,^6^ highlighting the critical link between cardiac metabolism, cardiac function, and heart regeneration. The adult mammalian heart is a very versatile organ from a metabolic point of view. Most (95%) of adult cardiac ATP is produced through mitochondrial oxidation of substrates, with fatty acids and glucose oxidation accounting for up to 60% of the mitochondrial ATP production.^7,8^ However, the heart is also able to oxidize ketonic bodies, amino acids, and lactate.^9^

In this review, we summarize the main metabolic pathways of the mammalian heart and how modulation of these metabolic pathways through genetic and pharmacological approaches influences cardiomyocyte proliferation and heart regeneration.

## Metabolic Dynamic of the Developing, Neonatal, and Adult Heart

Metabolism in the heart is a dynamic process that depends both on the life stage and pathophysiological status of the organism in addition to the nutrient availability. Heart development, especially in the early stages, occurs in low oxygen conditions.^10^ This, together with the low availability of fatty acids in the embryonic blood, plus glycolysis being a source of building blocks for proliferation and growth,^11,12^ causes the developing heart to rely mainly on glucose utilization versus fatty acid oxidation.^3^ This implies that the heart undergoes a metabolic shift from embryonic to adult life. Interestingly, an initial metabolic switch occurs during mid-gestation (Figure 1), which results in mitochondrial maturation, mitochondrial network development, and closure of the mitochondrial permeability transition pore. These mitochondrial changes are associated with enhanced cardiomyocyte maturation. However, the precise mechanistic link between mitochondrial maturation and cardiomyocyte differentiation is not well understood.^13,14^

This increase in mitochondrial oxidative metabolism may be controlled by a spatially and temporally defined loss of HIF1 signaling since gain-of-function of HIF1 pathway impairs cardiac maturation around E12.5 to E14.5.^15^ However, during the transition to postnatal life, the oxygenation level of the heart increases, mainly to the full separation of arterial and venous blood on cardiac shunts closure when the pulmonary respiration is initiated. Hypoxia fate-mapping studies have shown that the adult heart retains a hypoxic niche, formed by scattered cardiomyocytes through the whole organ.^16^ These cardiomyocytes, in addition to retaining a more fetal-like metabolic signature, also maintain the ability to proliferate and are the main contributors to cardiomyocyte turnover in the adult murine heart. Interestingly, chronic exposure of adult mice to low oxygen tensions (7%) results in increased cardiomyocyte proliferation, improved left ventricular ejection fraction (LVEF), and reduced fibrosis after myocardial infarction.^17^ Moreover, hypoxia exposure of human patients with prior myocardial infarction resulted in improved cardiac function, which was sustained for weeks following cessation of hypoxia exposure.^18^ Altogether, these findings highlight the importance of oxygen levels and hypoxia signaling in regulating cardiac metabolic networks and, ultimately, cardiomyocyte proliferation and cardiac regeneration.

## Fatty Acid Oxidation

In addition to the increased oxygen levels in the postnatal heart, a second and critical metabolic switch happens early after birth. The transition from the intrauterine low-oxygen environment to the oxygen-rich postnatal environment rewires cardiac metabolism, where fatty acid oxidation becomes the main energy source of the neonatal and adult heart instead of anaerobic glycolysis and glucose oxidation.^5^ However, the mitochondrial utilization of fatty acids also increases mitochondrial ROS production, resulting in DNA damage, activation of DNA damage response (DDR), and cell cycle exit.^5^ In fact, ROS measurements in vitro in neonatal rat ventricular myocytes showed that switching culture conditions from glucose media to fatty acid media increases the amount of ROS at the chromatin level.^19^ Thus, modulation of glucose versus fatty acid utilization in cardiomyocytes is directly linked to ROS production, DNA damage, and proliferative capacity of cardiomyocytes. Onset of mitochondrial fatty acid oxidation in the neonatal heart is also linked to their supply though breastfeeding. In fact, neonatal mice bred with fat-deficient milk mums (and on fat-free milk later on) display increased cardiomyocyte proliferation up to 10 weeks of age, after which compensatory fatty acid synthesis in the liver results in liver steatosis and blunts induction of cardiac proliferation.^20^

**Figure 2:**
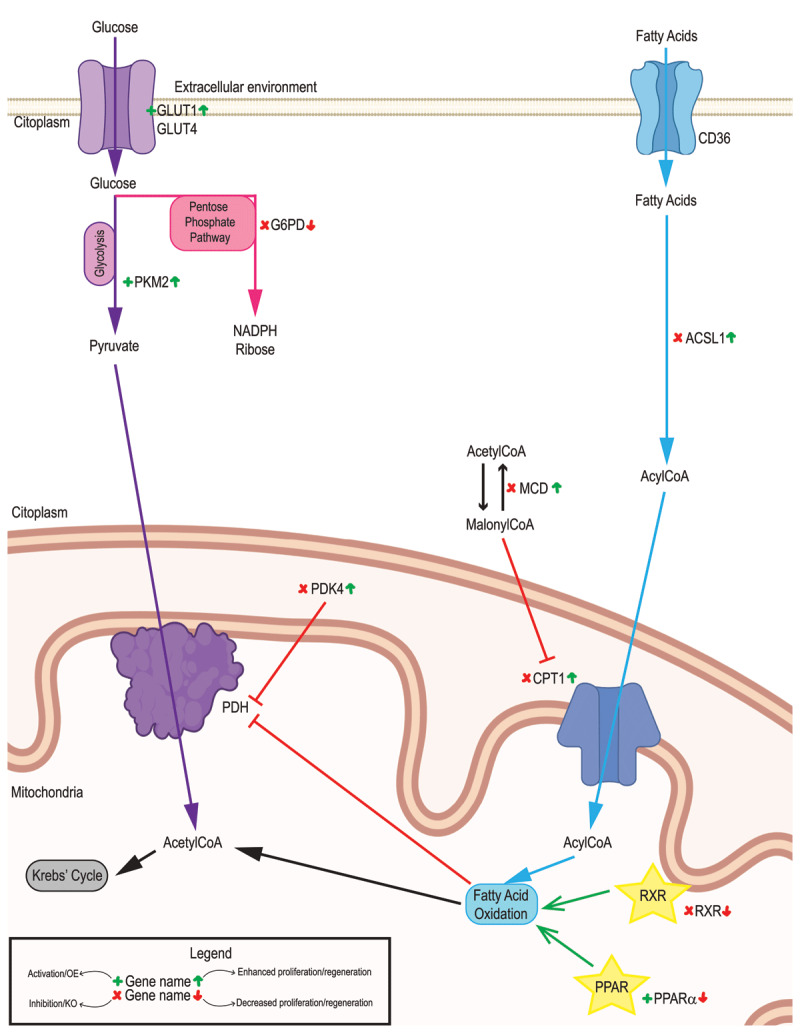
**Glucose and fatty acids metabolism in the adult heart.** Glucose oxidation (purple), fatty acids metabolism (blue), and Pentose Phosphate Pathway (pink) representation. Genetic/pharmacological models are indicated for activation/overexpression (green + symbol) or inhibition/deletion (red cross). Their outcomes are indicated on the right side of the name for increased (green up arrow) or decreased (red down arrow) proliferation/regeneration. Signaling pathways are indicated with a yellow star.

In order for the heart to utilize fatty acids, long-chain fatty acids enter the cardiomyocyte through the CD36 transporter.^21^ Once in the cytoplasm, they are bound to acetyl coenzyme A (acetyl CoA) through the action of acyl-CoA synthetase long-chain family member 1 (ACSL1), a rate-limiting step in fatty acid oxidation.^22^ Interestingly, neonatal cardiac-specific knockdown of ACSL1 in mice increased cardiomyocyte proliferation up to the first 2 months of life and improved cardiac function after myocardial infarction in adult mice.^23^ Acyl-CoAs enter the mitochondria by the action of the carnitine palmitoyltransferase 1 (CPT1). CPT1 activity increases at postnatal day 7, coincident with the loss of proliferative capacity in the mammalian neonatal heart.^24^ A recent report by Braun lab shows that inactivation of CTP1B in cardiomyocytes leads to increased proliferation. This effect is due to 2/oxoglutarate accumulation on, and subsequent activation of, the lysine demethylase KDM5. Activation of KDM5 decreased transcription and shifted cardiomyocytes to a less mature, more proliferative state,^25^ opening new connections between metabolism and myocardial proliferation through epigenetic regulation of proliferative genes. Interestingly, pharmacological inhibition of CPT1 using etomoxir reduced fatty acid oxidation and promoted cardiomyocyte proliferation in neonatal mice,^26^ but this was not sufficient for induction of adult cardiomyocyte proliferation (Figure 2).^27^

Fatty acid entry into the mitochondria is also inhibited by malonylCoA. Malonyl CoA decarboxylase (MCD) decreases the levels of malonylCoA and thus derepresses fatty acid oxidation. Cardiac-specific deletion of MCD decreases fatty acid oxidation, increases mitochondrial glucose oxidation, and improves cardiac function in mouse models of ischemia/reperfusion injury.^28^ In addition, pharmacological inhibition of MCD in the same model has similar outcomes.^29^

Fatty acid oxidation is regulated by the peroxisome-proliferator-activated receptor (PPAR) pathway,^30^ through the transcriptional activation of fatty acid oxidation enzymes and others through PPARα.^31^ Pharmacological inhibition of PPARα in mESCs impaired differentiation into cardiomyocytes.^32^ Moreover, PPARα agonist GW7647 administration in neonatal mice reduced cardiomyocyte proliferation by increasing cardiomyocyte hypertrophy and binucleation,^26^ indicating that promoting fatty acid oxidation reduces the proliferative capacity of the heart and, potentially, its regenerative capacity (Figure 2).

Despite these reports, the mechanistic link between fatty acid metabolism and cardiomyocyte maturation is not very clear. The recent report by the Braun group provided some elegant mechanistic insights. In addition, previous reports by our group suggest that fatty acid metabolism in cardiomyocytes directly causes chromatin DNA damage, with subsequent activation of DNA damage response. Downstream of DNA damage response, there are a number of mechanisms that can directly induce cardiomyocyte maturation. For example, the DDR kinase ATM regulates calcineurin expression.^33^ Calcineurin not only induces cardiomyocyte hypertrophy in response to stress but also regulates nuclear translocation of Hoxb13 which, together with Meis1, regulates a cardiomyocyte maturation program and induces a switch from hyperplastic to hypertrophic growth.^34^

## Glucose Metabolism

As outlined earlier, the loss of proliferative capacity of the neonatal hearts coincides with a metabolic switch from glucose utilization in favor of fatty acid oxidation. Glucose utilization, and specifically glycolysis, while less energetically efficient (2 ATP molecules versus 36 ATP molecules per molecule of glucose), provides the necessary building blocks and metabolic precursors to sustain cell proliferation. Indeed, highly proliferative cells show higher glycolytic rates than more quiescent cells.^12^ Glycolysis contributes to approximately 40% of neonatal cardiomyocyte ATP production while it drops to 5% of adult ATP production.^35^ Glucose transporters 1 and 4 are the main isoforms involved in the transport of glucose into the cardiomyocytes during neonatal and adult stages, respectively.^36^ Overexpression of GLUT1 promoted glycolysis and nucleotide synthesis and increased heart regeneration upon cryoinjury in juvenile and adult mice.^37^

Apart from the role of glucose uptake and glycolysis in cardiomyocyte proliferation, glycolytic enzymes can play a direct role in heart regeneration. For example, pyruvate kinase muscle isoforms (PKM) switch during postnatal cardiac maturation; while PKM2 is the main isoform expressed in neonatal hearts and is highly regulated by effectors and allosteric interactions, PKM1 is constitutively active and is the main adult isoform.^38^ Over-expression of PKM2 after myocardial infarction in mice increases cardiomyocyte proliferation and improves left ventricular systolic function, indicative of functional heart regeneration. Interestingly, this pro-proliferative effect of PKM2 is independent of glycolytic induction and instead is due to two parallel effects: activation of pentose phosphate pathway with subsequent increase in nucleotide synthesis and antioxidant defense through induction of glucose-6-phosphate dehydrogenase (G6PD) and cell cycle induction though β-catenin pathway activation.^38^ This study highlights not only the narrow interconnection between different central metabolic pathways but also that metabolic enzymes can play a role in cardiomyocyte proliferation that is totally independent of their metabolic functions. It also would be interesting to check whether other glycolytic enzymes display similar interactions with pro-proliferative pathways (Figure 2).

Glycolytic intermediates also are involved in other central metabolic pathways relevant to cardiomyocyte proliferation. For example, glucose-6-phosphate generated in the first step of glycolysis can be rerouted to the pentose phosphate pathway, a source of NADPH and ribose precursors for nucleotide synthesis.^39^ Remarkably, deletion of the rate-limiting enzyme of the pentose phosphate pathway, G6PD limits reduced glutathione (GSH) regeneration in the heart.^40^ This also could have potential implications in the ability of the heart to fight against mitochondrial ROS, thus impacting activation of DNA damage response.

Another glycolytic metabolite, fructose-6-phosphate, contributes to the hexosamine biosynthetic pathway. This pathway provides substrates for protein O-GlcNAcylation.^41,42^ Indeed, overexpression of O-GlcNAcase, which removes the N-acetylglucosamine residues from serine and threonine residues, impairs cardiomyocyte cell cycle entry in human iPSC-derived cardiomyocytes.^43^ This establishes a clear link between protein O-GlcNAcylation and cardiomyocyte proliferation and, potentially, a link between glycolytic intermediate levels and cardiomyocyte cell cycle regulation. Importantly, mitochondrial oxidation of fatty acids and glucose oxidation are not independent pathways. They are mutually regulated through the Randle Cycle:^44^ glucose uptake and utilization are reduced when fatty acid oxidation is intense. The reduction of glucose as an energy source is due to inhibition of mitochondrial pyruvate dehydrogenase by fatty acid oxidation-derived acetyl CoA, due to activation of pyruvate dehydrogenase kinase 4 (PDK4). Interestingly, cardiomyocyte-specific deletion of PDK4 in adult mice promoted glucose oxidation over fatty acid oxidation, increased cardiomyocyte proliferation, and enhanced heart regeneration and cardiac function after myocardial infarction (Figure 2).^20^

A similar approach of PDK inhibition was achieved by administration of the PDK inhibitor dichloroacetate, which resulted in increased glucose oxidation and increased response to reperfusion.^45,46^ Mechanistically, this is achieved by lower ROS production upon glucose utilization and thus a lower extent of activation of DNA damage response pathway. Similarly, while high succinate levels inhibit cardiomyocyte proliferation due to increased ROS production and increased oxidative DNA damage, inhibition of mitochondrial succinate dehydrogenase by malonate results in increased cardiomyocyte proliferation in neonatal mice and cardiac regeneration post-MI in both neonatal and adult mice.^47^ This indicates a pro-regenerative effect, not just cardioprotection. Interestingly, malonate administration and subsequent SDH inhibition results in a metabolic switch towards glycolysis. It also contributes to regeneration by promoting revascularization, suggesting an additional role of non-cardiomyocyte metabolism in heart regeneration.

## Amino Acid Metabolism

**Figure 3:**
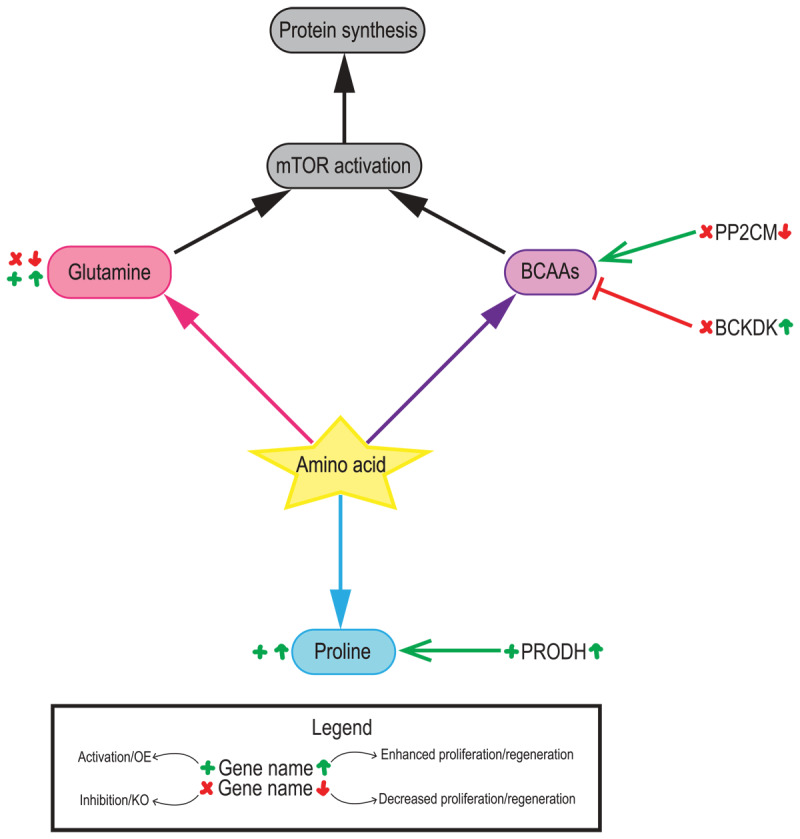
**Contributions of amino acid metabolism in the adult heart.** Contributions of glutamine (pink), branched-chain amino acids (BCAAs, purple) and proline (blue) to cardiac regeneration. Genetic/pharmacological models are indicated for activation/overexpression (green + symbol) or inhibition/deletion (red cross). Their outcomes are indicated on the right side of the name for increased (green up arrow) or decreased (red down arrow) proliferation/regeneration.

In the past decade, there has been an increasing interest in amino acids as a source of myocardial energy, specifically under stress and/or diseased conditions.^48,49^ These include alanine, glutamine/glutamate and the branched-chain amino acids valine, leucine and isoleucine.^50,51,52^ In addition, cardiomyocyte proliferation also requires increased protein synthesis and thus, increased amino acid metabolism. Altogether, these reports highlight the importance of amino acid metabolism as adaptative metabolic pathways in the heart (Figure 3).

Branched-chain amino acids account for only 2% of the total ATP production in the heart,^53^ but they have an important role in the regulation of critical signaling pathways, such as mTOR and insulin.^54^ Valine, leucine, and isoleucine abundance in cardiomyocytes increases progressively until postnatal day 9, and progressively decreases to levels equivalent to those at birth by postnatal day 23.^55,56^ Branched-chain amino acids, especially leucine, activate mTORC1 pathway and stimulate cell growth.^57^ Branched-chain amino acid metabolism is altered in pathological cardiac remodeling upon injury.^52,58^ Moreover, overexpression of the mitochondrial phosphatase 2C (PP2Cm), an activator of branched-chain amino acid catabolism, ameliorates oxidative stress in cultured H9C2 cells. However, implications of branched-chain amino acid levels and catabolism have not been explored in the context of cardiac proliferation and regeneration. Nevertheless, existing knowledge in cardiac injury models, such as pressure-overload or ischemia-reperfusion injury, suggest that inhibition of branched-chain amino acid catabolism and their accumulation could be detrimental for regeneration.^52,59,60^

Glutamine, the most abundant amino acid in the body, plays a critical role in nitrogen exchange in addition to pH regulation. Cardiac glutamine is metabolized through glutaminase (GLS) and converted into glutamate and ammonia. The generated glutamate can enter the tricarboxylic acid cycle and generate 2-oxoglutarate, a source of carbons for biosynthesis. Glutamate can also promote glutathione synthesis, a critical antioxidant molecule.

While the role of glutamine in cardiac regeneration needs further investigation, given the important link between ROS levels, DNA damage, and cardiomyocyte proliferation, glutamine metabolism could play a role in cardiomyocyte proliferation. Finally, in combination with leucine, increased levels of glutamine-derived glutamate activate mTOR pathway.^61^ Interestingly, glutamine catabolism, known as glutaminolysis, is upregulated in cardiomyocytes under oxidative stress, improving cell viability by maintaining ATP and gluthathione levels by anaplerosis.^62^ While the link between glutamine metabolism and cardiomyocyte proliferation and cardiac regeneration upon injury remains unknown, given the role that oxidative stress and ROS production play in cardiomyocyte cell-cycle exit, glutamine could be an interesting metabolic candidate to induce cardiac regeneration upon injury.

Proline is another amino acid that has recently received attention for its importance in cardiac metabolism. In vitro studies have shown that proline can reduce ROS production during oxidative stress. In addition, proline administration after myocardial infarction reduced infarct size and decreased oxidative damage through direct quenching of reactive oxygen species but also through regulation of mitochondrial metabolism.^63^ In this regard, proline catabolism seemed to be critical to maintain normal mitochondrial function and ATP production during hypoxia in cardiomyocytes.^64^ Interestingly, proline dehydrogenase (PRODH), the rate-limiting step of proline degradation, can donate electrons to the electron transport chain to generate ATP.^64^ In fact, our own results show that proline catabolism increased during hypoxia-induced cardiac regeneration.^17^ However, no current research provides a direct mechanistic link between proline degradation and cardiomyocyte proliferation.

Utilization of amino acids also requires the removal of the ammonia generated from their amino groups. Generally, this ammonia is eliminated as urea by the urea cycle. Almost 50 years ago, the heart was shown to display arginase activity, especially in infarcted areas, and to have increased urea production upon injury.^65^ Interestingly, the failing human heart was shown to have increased expression of urea transporters.^66^ In addition, early activation of mitochondrial and amino acid metabolism in embryonic hearts also increased the abundance of urea cycle metabolites.^67^ Despite these findings and the fact that blood levels of urea cycle metabolites are altered during cardiac pathologies, a deeper role of the urea cycle in the heart and its connections with cardiac regeneration remains unexplored, mainly due to the existing dogma of the urea cycle not being active in the heart. However, one cannot rule out the notion that the urea cycle could be transiently upregulated in the heart in contexts where amino acid utilization is increased.

## Ketone Body Metabolism

The main ketone bodies (acetoacetate and 3-beta-hydroxybutyrate) are produced in the liver and are used by the heart as energy substrates when other substrates are not readily available.^68^ In fact, failing hearts have increased utilization of ketone bodies.^69^ However, it has been recently proposed that the myocardial utilization of ketone bodies is proportional to their blood levels: when concentrations of 3-beta-hydroxybutyrate rise over 2mM, it becomes the main source of cardiac energy even in the presence of normal levels of fatty acids and glucose.^70^

Not much is known about the utilization of ketone bodies in the postnatal stages. However, some studies have shown that ketone bodies’ catabolism is activated during the regenerative stages of the murine neonatal heart.^71^ In fact, overexpression of 3-hydroxy-3-methylglutaryl-CoA synthase 2, which catalyzes a rate-limiting step in ketogenesis, improved cardiac function in a mouse model of myocardial infarction and increased the number of proliferative cardiomyocytes.^72^ Moreover, dietary administration or infusion of 3-beta-hydroxybutyrate in mice and dogs improved left ventricular function and remodeling.^73^ Overall, while ketone bodies have been recently shown to have beneficial effects during heart failure, the mechanism involved in this effect remains unknown.^74^

## Conclusion

Cardiac metabolism is a flexible and dynamic process that regulates numerous aspects of cardiac physiology and pathology. The increased interest in metabolic adaptations occurring in the developing and adult hearts and their impact on cardiomyocyte cell cycle opens new venues for the development of new regenerative therapeutics.

## Key Points

- The heart switches from glucose-based metabolism in the embryo to fatty acid oxidation in the adult life.
- Cardiac metabolism directly impacts the proliferative capacity of cardiomyocytes through DNA damage and other epigenetic mechanisms.
- In general, promotion of glucose utilization over fatty acid oxidation in the adult heart restores cardiomyocyte proliferation and enhances the regenerative capacity of the heart.
- Due to the high metabolic flexibility of the adult heart, other nutrients, such as amino acids and ketone bodies, can also play a role in the regulation of cardiomyocyte cell cycle.
